# Image Retrieval Using the Fused Perceptual Color Histogram

**DOI:** 10.1155/2020/8876480

**Published:** 2020-11-24

**Authors:** Guang-Hai Liu, Zhao Wei

**Affiliations:** College of Computer Science and Information Technology, Guangxi Normal University, Guilin 541004, China

## Abstract

Extracting visual features for image retrieval by mimicking human cognition remains a challenge. Opponent color and HSV color spaces can mimic human visual perception well. In this paper, we improve and extend the CDH method using a multi-stage model to extract and represent an image in a way that mimics human perception. Our main contributions are as follows: (1) a visual feature descriptor is proposed to represent an image. It has the advantages of a histogram-based method and is consistent with visual perception factors such as spatial layout, intensity, edge orientation, and the opponent colors. (2) We improve the distance formula of CDHs; it can effectively adjust the similarity between images according to two parameters. The proposed method provides efficient performance in similar image retrieval rather than instance retrieval. Experiments with four benchmark datasets demonstrate that the proposed method can describe color, texture, and spatial features and performs significantly better than the color volume histogram, color difference histogram, local binary pattern histogram, and multi-texton histogram, and some SURF-based approaches.

## 1. Introduction

In the fields of image retrieval, pattern recognition, computer vision, and digital image processing, mimicking human cognition remains a challenge. In the human visual system, the perception of color begins with three types of cones in the retina called the *L*, *M*, and *S* cones. These contain pigments with different spectral sensitivities that produce trichromatic color sensations [1]. The LMS color space represents the response of the three types of cone photoreceptors in the human eye and can be translated into other color spaces or models. This allows the opponent and HSV color spaces to be calculated easily. Then, the question arises: how are opponent and HSV color spaces used to extract visual features for image retrieval? A multi-stage color model that combines the three-photoreceptors model with the opponent theory has been suggested [1]. The neural signals from the three cone photoreceptors of the eye are combined into opponent color channels at the retinal level and then transmitted to the brain. Thus, it is possible to utilize a multi-stage color model to describe and represent an image for image retrieval.

In previous work, we have proposed color difference histograms (CDHs) [2] to image retrieval based on the CLE L^*∗*^a^*∗*^b^*∗*^ color space [2]. However, the traditional CIE color difference formula was originally designed for simple color patches in controlled viewing conditions and is not adequate for computing image differences for spatially complex image stimuli [3]. In this paper, we improve and extend the CDH method using a multi-stage model to extract and represent an image in a way that mimics human perception.

Our main contributions are as follows: (1) a novel visual feature descriptor is proposed to represent an image. It has the advantages of a histogram-based method and is consistent with visual perception factors such as spatial layout, intensity, edge orientation, and the opponent colors. (2) We improve the distance formula of CDHs; it can effectively adjust the similarity between images according to two parameters. The proposed method provided efficient performance in similar image retrieval rather than object searching.

The rest of this paper is organized as follows.  Section 2 reviews image retrieval techniques from literature published in recent decades, while Section 3 describes the fused perceptual color histogram. We describe CBIR experiments in Section 4; then Section 5 concludes the paper.

## 2. Related Work

The widely used attributes used to represent image content are color, texture, and shape features. In the MPEG-7 standard, different methods have been proposed to describe these features and different descriptors have been applied in CBIR. The color histogram is a widely used method to describe color features. It is invariant to orientation and scale and can give powerful image classification potential. For this reason, and for its simplicity and effectiveness, color descriptors were very popular in the early days of CBIR. The color descriptors used in the MPEG-7 standard include the dominant color descriptor, color layout descriptor, color structure descriptor, and scalable color descriptor [4]. Those color descriptors aim to provide a compact color description, capture the spatial distribution of color, and express the local color structure. Several other color features have also been proposed. Color volume and color difference have been utilized to extract color features [2, 5] and for saliency detection [6]. Varior et al. proposed the learning of invariant color features for person re-identification [7].

Texture descriptors can be used to characterize repeated geometric patterns or color regions. In the MPEG-7 standard, texture descriptors include the homogeneous texture descriptor, texture browsing descriptor, and edge histogram descriptor[4]. In recent decades, many texture analysis methods have been proposed, including Haralick's gray co-occurrence matrix (GLCM) features [8], the Markov random field (MRF) model [9], and local binary patterns (LBP) [10]. In recent years, many algorithms have been proposed for combining multiple visual cues to improve discriminative power. Liu et al. proposed the texton-based methods for image retrieval [11–13]. Singh et al. proposed a color texture descriptor based on local binary patterns for color image retrieval [14]. In order to capture the evolution of repeated geometric patterns, Thompson et al. proposed the edge-local binary pattern (edgeLBP) technique for 3D object retrieval and classification [15]. Dubey et al. proposed a multichannel decoded LBP method and utilized it for CBIR [16]. Roberto et al. proposed the orthogonal moments for texture classification [17]. A set of Gabor filters with different frequencies and orientations can mimic the perception of the human visual system (HVS). It is helpful for extracting useful visual features from an image for various applications. Based on saliency cues, bar-shaped structures, and Gabor filters, Liu et al. proposed the salient-structure histograms to image retrieval and achieved excellent performance [18].

Shape plays an important role in understanding and identifying objects; however, it is difficult to extract shape features. In many cases, shape feature extraction is often performed via accurate segmentation, which is a very difficult issue in image processing. In the MPEG-7 standard, the region-based shape descriptor, contour-based shape descriptor, and 3D shape descriptor are considered to provide good approximations of segmentation. These descriptors can describe the regions, contours, and shapes of 2D images and 3D volumes. In order to avoid accurate segmentation, some local feature descriptors, including scale-invariant feature transform (SIFT) descriptors [19], and the histograms of oriented gradients (HOG) [20], are also used in shape matching and recognition. Hong et al. proposed a novel shape descriptor that characterizes the local shape geometry based on integral kernels with respect to the size of the shape at a range of feature scales [21]. Clement et al. proposed a structural object description by learning spatial relations and shapes and utilized it for object recognition [22]. Žunić et al. introduced a disconnectedness measure for multi-component shapes [23]. Liu et al. proposed a novel structured optimal graph based sparse feature extraction method for learning the local discriminative information [24]. Malu et al. proposed a dynamic circular mesh-based shape and margin descriptor to combine the functions of structural and global contour-based descriptors and utilized it for object detection [25]. Mehmood et al. have extended the local feature descriptors for image retrieval by using the visual words model [26–29].

In the last decade, deep learning, especially by convolutional neural networks (CNNs), has been successfully applied to a variety of domains [30–38]. It requires a mass of data for training and provides useful information for various applications, including image retrieval and pattern recognition. CNN-based methods use a pre-trained or fine-tuned CNN to extract features for image retrieval and classification, for instance, by extraction of global features from its fully connected layer and extraction of local features from its intermediate layer [30–34]. Discovering how to combine deep learning with large amounts of data could provide computers with human-like image recognition capabilities. However, this field is immature and many challenges remain.

In this paper, we propose a simple yet efficient image retrieval method that simulates the dual-stage model of color vision to mimic human color perception.

## 3. The Fused Perceptual Color Histogram

Feature extraction has a close relationship to color space. In digital image processing, the RGB color space is very popular for representing color but has two obvious shortcomings: (1) it is not directly based on the cones in the human eye and (2) it is not uniform with respect to human color perception. In previous work, we proposed using color difference histograms (CDHs) [2] in image retrieval. The unique characteristic of CDHs is the way they count the perceptually uniform color differences between two points with different backgrounds with regard to colors and edge orientations in the CLE L^*∗*^a^*∗*^b^*∗*^ color space [2]. However, the traditional CIE color difference formula was originally designed for simple color patches in controlled viewing conditions and is not adequate for computing image differences for spatially complex image stimuli [3].

**Figure 1:**
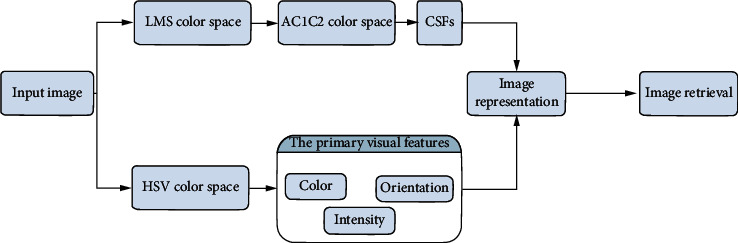
Flow diagram of the proposed feature extraction and discriminative representation system within the CBIR framework.

In this paper, we improve and extend the CDH method by utilizing a dual-stage model to extract and represent an image in a way that mimics human perception. We propose a novel visual descriptor based on fused perceptual color information by using the attributes of the opponent color and HSV color spaces. It aims to represent image content using intensity, color, and edge orientation features in the opponent color and HSV color spaces, giving it the power to describe color, texture, edge, and spatial features.  Figure 1 illustrates the proposed feature extraction and discriminative representation system within the CBIR framework, which is composed of three parts: (1) RGB color space conversion into other color spaces, including XYZ, LMS, and HSV, (2) primary visual feature calculations from the HSV color space, and (3) image representation and image retrieval.

### 3.1. LMS and Opponent Color Spaces

In the trichromatic theory of human color vision, it is suggested that there are three kinds of cone cells (also called photoreceptors) with different spectral sensitivities. Signals produced by the three photoreceptors are sent to the central nervous system and perceived as color sensations [1]. In normal human trichromacy, the three kinds of photoreceptors having peak sensitivities in the large-, medium-, and short-wavelength portions of the visible spectrum are called the *L*, *M*, and *S* cones, respectively [3].

In order to make good use of perceptual color information, we first convert the original color image from the RGB color space to the LMS space in two steps. The first is a conversion from RGB to XYZ tristimulus values, which is a device-independent color space. This conversion can be calculated as follows [3]:

(1) $$\begin{array}{c} \left[\begin{array}{c} X\\ Y\\ Z \end{array}\right]=\left[\begin{array}{ccc} 0.5141 & 0.3239 & 0.1604\\ 0.2651 & 0.6702 & 0.0641\\ 0.0241 & 0.1228 & 0.8444 \end{array}\right]\left[\begin{array}{c} R\\ G\\ B \end{array}\right]\text{.} \end{array}$$

In the device-independent *XYZ* space, we can convert the original image from the *XYZ* to *LMS* color space using the following conversion [3]:

(2) $$\begin{array}{c} \left[\begin{array}{c} L\\ M\\ S \end{array}\right]=\left[\begin{array}{ccc} 0.3897 & 0.6890 & -0.0787\\ -0.2298 & 1.1834 & 0.0464\\ 0.0000 & 0.0000 & 1.0000 \end{array}\right]\left[\begin{array}{c} X\\ Y\\ Z \end{array}\right]\text{.} \end{array}$$

In the *LMS* color space, a great deal of skew is shown in the data. In order to largely eliminate this skew, we can convert the data to a logarithmic space [3]:

(3) $$\begin{array}{c} \left\{\begin{array}{c} L=\log\;L\text{,}\\ M=\log\;M\text{,}\\ S=\log\;S\text{.} \end{array}\right. \end{array}$$

The large-, medium-, and short-wavelength cone signals (*LMS*) are combined to form a variant of the opponent color model called the *AC*_1_*C*_2_ opponent color space [39], which is calculated as follows:

(4) $$\begin{array}{c} \left[\begin{array}{c} A\\ C_{1}\\ C_{2} \end{array}\right]=\left[\begin{array}{ccc} 0.990 & -0.106 & -0.094\\ -0.669 & 0.742 & -0.027\\ -0.212 & -0.354 & 0.911 \end{array}\right]\left[\begin{array}{c} L\\ M\\ S \end{array}\right]\text{.} \end{array}$$

After the above calculations or conversions, we utilize the *LMS* and *AC*_1_*C*_2_ opponent color spaces to extract visual features by using filters that approximate the contrast sensitivity functions (CSFs) of the human visual system [40].

### 3.2. HSV Color Space

The HSV color space can be represented as a cylindrical coordinate system in which H, S, and V are its three coordinate variables. Variable H signifies the hue, which represents the perceived colors red, yellow, green, and blue, or a combination of two of them [41]. Saturation (S) refers to the relative purity or degree to which the color is combined with white, and Value (V) indicates the brightness relative to a similarly illuminated white color [41, 42].

**Figure 2:**
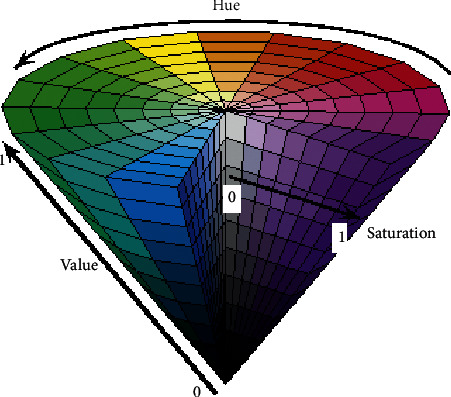
Illustration of the HSV color space [43].

In the HSV cylindrical coordinate system [42], H represents the angle of rotation and ranges from 0 to 360 degrees; S represents the size of the radius (range = 0‐1); and V indicates the height of the cylinder (range = 0‐1), as shown in Figure 2.

### 3.3. Feature Quantization

In this section, color, edge orientation, and intensity maps are utilized to extract visual features via the feature quantization technique. The HSV color space is widely utilized in the field of image retrieval, is consistent with human visual perception, and can better describe the content of an image. Therefore, the color, edge orientation, and intensity features are extracted in HSV color space.

The quantized color comes from various combinations of the H, S, and V components. In our method, the H component is uniformly quantized into 6 bins, and both S and V components are uniformly quantized into 3 bins, resulting in the color map *C*(*x*, *y*) = *ω*, *ω* ∈ {0,1,…, *N*_*C* _ − 1} and *N*_*C*_ = 54.

Compared with the color and edge orientation extraction method utilized in CDHs [2], the Sobel operator is a convenient and simple edge detector. Edge orientation is first extracted from the V component using the Sobel operator and then uniformly quantized into *N*_*O*_=36 bins. We denote the edge orientation map as *O*(*x*, *y*)=*ε*, *ε* ∈ {0,1,…, *N*_*O*_ − 1}.

Here, the intensity map *I*(*x*, *y*)  is obtained directly by uniformly quantizing the V component, where  *I*(*x*, *y*)=*τ*, *τ* ∈ {0,1,…, *N*_*I*_ − 1} and  *N*_*I*_=16 .

### 3.4. Approximating the Contrast Sensitivity Functions (CSFs)

The human visual system is much less sensitive to colors at high frequencies than at low ones. Hence, using contrast sensitivity functions (CSFs) to modulate frequencies that are less perceptible can better simulate the human visual system [39, 40]. In this paper, the CSFs are first used to remove information that is invisible to the human visual system in the opponent color space *AC*_1_*C*_2_.

The *AC*_1_*C*_2_ color space is spatially filtered using the CSFs by computing the difference between two points under various backgrounds in terms of colors, edge orientations, and intensity. Each channel in *AC*_1_*C*_2_ is spatially filtered by using the CSFs to approximate the human visual system, as expressed in formulas (5) and (6) [39].

(5) $$\begin{array}{c} E_{i}=e^{-\left(x^{2}+y^{2}\right)/\sigma_{i}^{2}}\text{,} \end{array}$$

(6) $$\begin{array}{c} \left\{\begin{array}{c} A\prime =\frac{\sum_{i\in \left[1,2,3\right]}w_{i}\left(A⊗E_{i}\right)}{3}\text{,}\\ C_{1}^{\prime }=\frac{\sum_{i\in \left[1,2\right]}w_{i}\left(C_{1}⊗E_{i}\right)}{2}\text{,}\\ C_{2}^{\prime }=\frac{\sum_{i\in \left[1,2\right]}w_{i}\left(C_{2}⊗E_{i}\right)}{2}\text{.} \end{array}\right. \end{array}$$

The weights (*w*_*i*_)  and spreads (*σ*_*i*_) of the CSFs are listed in Table 1. The filtered opponent color space is denoted as *A*′*C*_1_′*C*_2_′. Here, we utilize *A*′*C*_1_′*C*_2_′ to represent features, together with color, edge orientation, and intensity maps.

### 3.5. Feature Representation

Let there be two-pixel locations (*x*, *y*) and (*x*′, *y*′) with *d* as the spacing distance. Then, the feature representation of *C*(*x*, *y*),  *O*(*x*, *y*) , and *I*(*x*, *y*) can be expressed as follows:

(7) $$\begin{array}{c} H_{c}\left[C\left(x,y\right)\right]=\left\{\begin{array}{c} \overset{M-1}{\underset{x=1}{\sum }}\overset{N-1}{\underset{y=1}{\sum }}\sqrt{\Delta A^{2}+\Delta C_{1}^{2}+\Delta C_{2}^{2}\text{,}}\\ \text{where }C\left(x,y\right)=C\left(x^{\prime },y^{\prime }\right)\text{,} \end{array}\right.\;\\ H_{o}\left[O\left(x,y\right)\right]=\left\{\begin{array}{c} \overset{M-1}{\underset{x=1}{\sum }}\overset{N-1}{\underset{y=1}{\sum }}\sqrt{\Delta A^{2}+\Delta C_{1}^{2}+\Delta C_{2}^{2}\text{,}}\\ \text{where }O\left(x,y\right)=O\left(x_{i},y_{i}\right)\text{,} \end{array}\right.\\ H_{I}\left[I\left(x,y\right)\right]=\left\{\begin{array}{c} \overset{M-1}{\underset{x=1}{\sum }}\overset{N-1}{\underset{y=1}{\sum }}\left|\Delta A\right|\text{,}\\ \text{where }I\left(x,y\right)=I\left(x^{\prime },y^{\prime }\right). \end{array}\right. \end{array}$$

In the above formulas, *M* and *N* are the width and height of the image, respectively, Δ*A* = *A*′(*x*, *y*) − *A*′(*x*′, *y*′),  Δ*C*_1_ = *C*_1_′(*x*, *y*) − *C*_1_′(*x*′, *y*′), and  Δ*C*_2_ = *C*_2_′(*x*, *y*) − *C*_2_′(*x*′, *y*′). According to the above representations, the fused perceptual color histogram *H* can be obtained by concatenation of *H*_*c*_[*C*(*x*, *y*)], *H*_*o*_[*O*(*x*, *y*)], and *H*_*I*_[*I*(*x*, *y*)] as follows:

(8) $$\begin{array}{c} H=\text{CONCA}\left\{H_{c},H_{o},H_{I}\right\}\text{,} \end{array}$$

where CONCA{·} denotes the concatenation of *H*_*c*_, *H*_*o*_, and *H*_*I*_. The fused perceptual color histogram *H* contains the perceived color information related to the color, edge orientation, and intensity maps.

## 4. Experimental Results

In this section, we verify the effectiveness of the proposed method on four benchmark datasets containing more than 20,000 natural images, including Corel-5K, Corel-10K, Oxford buildings, and INRIA Holidays datasets. Image matching is adopted based on an improved distance formula of CDHs. For fair comparison, the proposed method will be compared with the current image retrieval methods, HOG [20], LBP [10], MTH [12], CDHs [2], CVH [5], BOW [38], and other methods [43, 44]. Most of these methods were developed for image retrieval. The details of the comparison methods are shown as follows:

(1) The codebook size for Bow was set as *K* = 1000 using the standard K-means clustering, and the cosine metric was used as the baseline of the Bow method; the local features are represented by the SIFT descriptors [20]. An LBP histogram with a dimensional feature vector of 256 bins and using the average values for the three-channel LBP histogram. The histogram of oriented gradients (HOG) feature descriptor is not a global image representation method; there are nine bins with a block size of three and a cell size of six. The L1 distance was adopted as the similarity measure of LBP histogram and HOG method. (2) CDHs [2], MTH [12], and CVH [5] follow the original setting of image representation and similarity measures. (3) The results of other methods [43,44] come from their conference reports.

### 4.1. Datasets

In the field of image retrieval, Corel datasets are the most widely utilized. Many algorithms have been used in CBIR experiments on Corel datasets for comparison. In this paper, we implemented a CBIR experiment with two Corel datasets: Corel-5K and Corel-10K. The Corel-5K dataset is a subset of the Corel-10K dataset and contains 50 classes. Each category includes 100 images sized 192 × 128 or 128 × 192 pixels in JPEG format. The Corel-10K dataset contains richer image content in 100 categories with 100 images in each category.

For comparisons of instance retrieval methods, we also evaluate our method on the Oxford5k and Holidays datasets. The Oxford5k contains 5,062 images which have 11 different Oxford landmarks. Each landmark is represented by 5 possible queries, and it leads to a set of 55 queries, over which an object retrieval system can be evaluated. The Holidays dataset has 1,491 images which contains 500 queries and 991 corresponding relevant images.

### 4.2. Distance Metrics

After feature extraction, the matching of images using distance metrics is a very important part of image retrieval. In previous work with CDHs [2], a new distance formula was proposed by expanding the Canberra distance. In this paper, we improve the distance formula of CDHs. Let *T* = {*T*_*i*_}_1_^*K*^ and *Q* = {*Q*_*i*_}_1_^*K*^ be the *K*-dimensional feature vectors of a template image and query image, respectively. The distance between them is simply calculated as follows:

(9) $$\begin{array}{c} D\left(T,Q\right)=\sum_{i=1}^{K}\frac{\left|Q_{i}-T_{i}\right|}{\left|Q_{i}+w_{1}\cdot T_{i}+w_{2}\cdot \mu_{T}\right|}\text{,} \end{array}$$

where *μ*_*T*_ is the mean of *T* and *w*_1_ and *w*_2_ are weight parameters used to enhance the difference between small bins and reduce the difference between large bins. In this paper, *K* is set to 106 bins, *w*_1_ = 1.4, and *w*_2_ = 0.2.

### 4.3. Performance Measures

All images were sampled for use as query images in each Corel dataset. Performance was evaluated using the average results of each query in terms of precision and recall. They are the most common performance evaluation criteria used in CBIR. They are defined as follows [2, 12, 13, 18]:

(10) $$\begin{array}{c} \text{Precision}=\frac{I_{N}}{N}\text{,}\\ \text{Recall}=\frac{I_{N}}{M}\text{,} \end{array}$$

where *I*_*N*_ is the number of images retrieved in the top *N* positions that are similar to the query image, *N* is the total number of images retrieved, and *M* is the total number of images in the dataset that are similar to the query image. Here, we set *N*=12 and *M*=100.

On the Oxford5k and Holidays datasets, mean average precision (mAP) is utilized to evaluate the performance of FPCH and other compared algorithms [43, 44]. We are following the original setting of query images and the corresponding relevant images.

### 4.4. Retrieval Performance and Discussion

In the proposed method, color, edge orientation, and intensity are utilized in representation, and their quantization number determines the vector dimensionality. Lower vector dimensionality not only is beneficial to rapid image retrieval but also requires less computation. Therefore, in the experiments, the quantization number of the above visual features needs to be determined and evaluated. We then investigate the influence of the feature quantization number.

**Figure 3:**
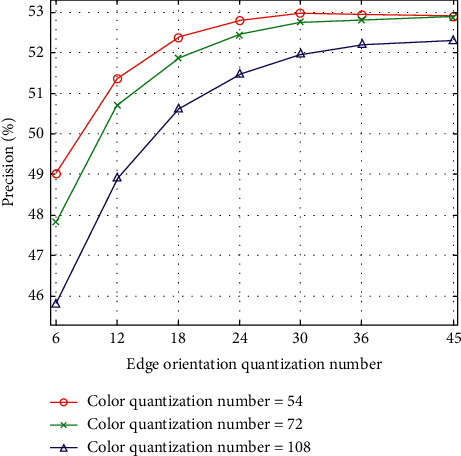
CBIR precision according to quantization numbers of color and edge orientation (intensity quantization number = 16).

**Figure 4:**
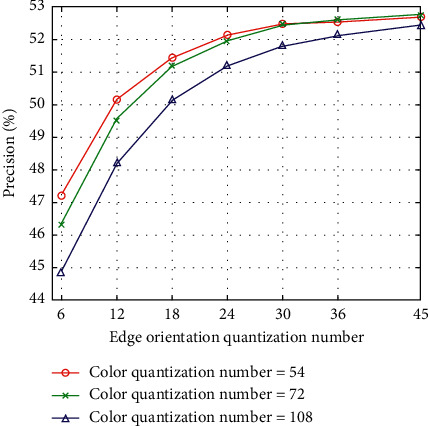
CBIR precision according to quantization numbers of color and edge orientation (intensity quantization number = 32).

**Figure 5:**
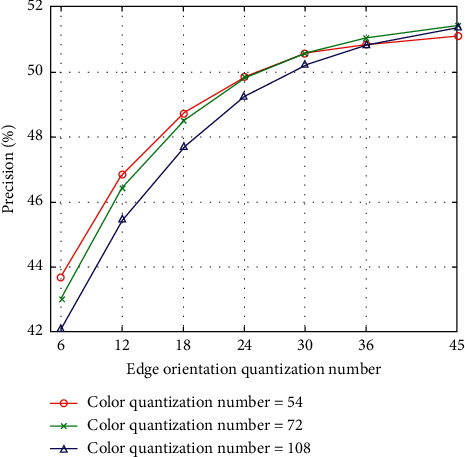
CBIR precision according to quantization numbers of color and edge orientation (intensity quantization number = 64).

The color quantization number consists of H, S, and V values. We set H to 6, 8, and 12, while S and V are fixed to 3. Hence, the color quantization number has 54 bins, 72 bins, and 108 bins. Furthermore, the quantization number of edge orientation has 6 bins, 12 bins, 18 bins, 24 bins, 30 bins, 36 bins, and 45 bins. The quantization number of intensity has 16 bins, 32 bins, and 64 bins. The experimental results for the Corel-10K dataset are shown in Figures 3–5. There is an evident phenomenon where the precision always increases as the edge orientation quantization number increases. When the intensity quantization number is 16 bins, the precision is inversely proportional to the color quantization number. However, the precision is proportional to the color quantization number when the intensity quantization numbers are 32 bins and 64 bins. In total, the precision decreases as the intensity quantization number increases. In order to balance the performance of the proposed algorithm with the number of feature vector dimensions, we ultimately choose a color quantization number of 54 bins, an edge orientation quantization number of 36 bins, and an intensity quantization number of 16 bins, resulting in a feature vector with a total of 106 dimensions.

**Figure 6:**
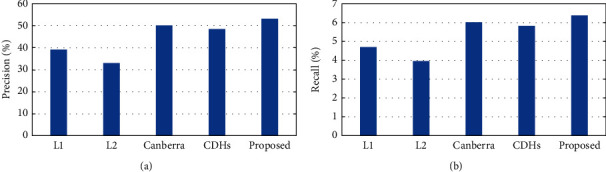
Performance comparison of distance formulas in (a) precision and (b) recall.

In order to illustrate the validity of the distance formula proposed in this paper, we compare its performance with the typical L1, L2, Canberra, and CDHs distance formulas in experiments on the Corel-10K dataset. The experimental results are shown in Figure 6, which shows that the proposed distance formula has the best performance. The Canberra, CDHs, and proposed distance formulas can all be regarded as weighted L1 distances with different weights. This weight can reduce the influence of differences between large bins in the histograms. The Canberra distance simply utilizes the reciprocal of the bin of the template and query images as the weight. The CDHs distance formula adds the mean of the template and query images based on the Canberra distance, while the proposed distance formula utilizes two parameters to adjust the weight accurately. Thus, the proposed distance formula can achieve better results.

In order to validate the performance of the proposed FPCH method, we compare it with CDHs [2], LBP [10], MTH [12], CVH [5], HOG [20], and BOW [38]. The experimental results are shown in Table 2. It can be seen that the precision of the proposed FPCH method is higher than those of LBP, MTH, CDHs, and CVH by 19.68%, 13.52%, 6.27%, and 3.37% on the Corel-5K dataset, respectively.

On the Corel-10K dataset, the precision of the proposed FPCH method is higher than BOW, HOG, LBP, MTH, CDHs, and CVH by 22.83%, 25.24%, 15.96%, 12.32%, 7.95%, and 4.61%, respectively. The recall of the proposed FPCH method is higher than those of the above methods on both datasets.

BOW and HOG are two typical methods of image retrieval based on object recognition; LBP and MTH are two different types of texture analysis methods, while LBP focuses on describing the spatial structure of texture. MTH represents the texton attributes of images through a series of fixed-size blocks with a certain number of identical pixels. Both CDHs and CVH can simulate human color perception. The proposed FPCH method can fuse the perceptual color information of the opponent color and HSV color spaces and can represent an image through edges, spatial structure, and texture information. Experiments on the Corel-5K and Corel-10K datasets show that the proposed FPCH method is superior to the above methods.

On the Holidays and Oxford5K datasets, we compared the FPCH method with some key-point-based or local-feature-based methods, including the extension or combination of SURF, VLAD, SOP, and RootHSV [44], and the HeW method using deep Conv layer of VGG16 [43].

As can be seen from Table 3, the proposed FPCH method is superior to the 8-SURF, 64-SURF, 4-RootHSV-L1, and 4-RootHSV-L2 methods [44]. However, the mAP of the proposed FPCH method is lower than that of HeW using deep Conv layer of VGG16 [43]. According to the results of Table 4, the proposed FPCH method is completely unsuitable for object searching.

**Figure 7:**
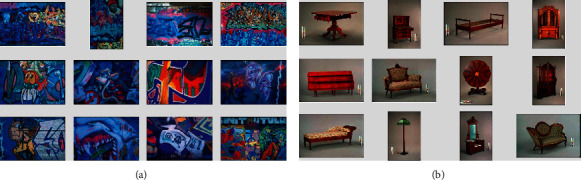
Two query examples from the Corel-5K and Corel-10K datasets: (a) graffiti and (b) furniture.

In order to visualize the retrieval effect of the proposed FPCH method, two images from the Corel-5K and Corel-10K datasets were selected as query images. The retrieval results are shown in Figures 7(a) and 7(b), where the top-left image is the query and 12 images were retrieved. It is worth noting that images of graffiti have rich color changes and images of furniture have significant differences on both sides of the objects' edges. It is clear that the proposed FPCH method can mimic human color perception and considers the differences between color, edge, and achromatic features. Therefore, 12 images similar to the query image can be correctly retrieved. Two retrieval examples are used to show the visual effects of low-level features rather than whether or not the performance is good since not all images can provide such a good effect.

### 4.5. Limitations of the Proposed Method

Although the proposed method not only approximates the contrast sensitivity functions (CSFs) of the human visual system to filter out information that is invisible to humans but also utilizes color, edge orientation, and intensity to represent and describe image features, the major limitation of the proposed method is that it cannot extract the local features and the high-level features. It is clear that the proposed FPCH method is entirely unsuitable for object searching according to the results of Table 4. Combining the high-level features with low-level features based on deep learning techniques will be studied in the future.

## 5. Conclusions

In this paper, we improve and extend the CDH method by utilizing a multi-stage model to extract and represent an image in a way that mimics human perception. We have proposed an image retrieval method that combines the attributes of the opponent color and HSV color spaces, namely, the fused perceptual color histogram. It aims to represent image content using intensity, color, and edge orientation features in the opponent color and HSV color spaces, allowing it to describe color, texture, edge, and spatial features.

In this process, we not only approximate the contrast sensitivity functions (CSFs) of the human visual system to filter out information that is invisible to humans but also utilize color, edge orientation, and intensity to represent and describe image features. The results of the experiments have shown that the proposed method can effectively describe the color, texture, and spatial structure of images and achieves better performance than existing techniques such as LBP, CDHs, CVH, 8-SURF, and 64-SURF methods on Holidays dataset.

The proposed method provides efficient performance in similar image retrieval rather than object searching.

## Figures and Tables

**Table 1:** Parameters of the CSFs [39].

Filters		Weight (*w*_*i*_)	Spread (*σ*_*i*_)
*A*(Achromatic)	*i* = 1	1.00327	0.0500
	*i* = 2	0.11442	0.2250
	*i* = 3	−0.11769	7.0000

*C *_1_(Red-green)	*i* = 1	0.61673	0.0685
	*i* = 2	0.38328	0.8260

*C *_2_(Blue-yellow)	*i* = 1	0.56789	0.0920
	*i* = 2	0.43212	0.6451

**Table 2:** Performance comparison of various CBIR methods.

Dataset	Performance	Method						
		BOW	HOG	LBP histogram	MTH	CDHs	CVH	FPCH
Corel-5K	Precision (%)	—	—	43.82	49.98	57.23	60.13	63.50
	Recall (%)	—	—	5.26	6.00	6.87	7.21	7.62

Corel-10K	Precision (%)	30.36	27.95	37.23	40.87	45.24	48.58	53.19
	Recall (%)	3.64	3.35	4.47	4.91	5.43	5.83	6.38

**Table 3:** Performance comparison of various CBIR methods on the Holidays dataset.

Methods	Dimension	mAP
8-SURF [44]	128	0.463
64-SURF [44]	128	0.625
4-RootHSV-L1 [44]	128	0.657
4-RootHSV-L2 [44]	128	0.675
HeW [43]	512	0.884
FPCH	106	0.699

**Table 4:** Performance comparison of various CBIR methods on the Oxford5K dataset.

Methods	Dimension	mAP
VLAD + SOP + 2-step + 8-SURF	1024	0.267
VLAD + SOP + 2-step + 64-SURF	4608	0.416
HeW [43]	512	0.728
FPCH	106	0.103

## Data Availability

The data and code are available at http://www.ci.gxnu.edu.cn/cbir/Dataset.
